# If We Share Data, Will Anyone Use Them? Data Sharing and Reuse in the Long Tail of Science and Technology

**DOI:** 10.1371/journal.pone.0067332

**Published:** 2013-07-23

**Authors:** Jillian C. Wallis, Elizabeth Rolando, Christine L. Borgman

**Affiliations:** Department of Information Studies, Graduate School of Education and Information Studies, University of California Los Angeles, Los Angeles, California, United States of America; Northwestern University, United States of America

## Abstract

Research on practices to share and reuse data will inform the design of infrastructure to support data collection, management, and discovery in the long tail of science and technology. These are research domains in which data tend to be local in character, minimally structured, and minimally documented. We report on a ten-year study of the Center for Embedded Network Sensing (CENS), a National Science Foundation Science and Technology Center. We found that CENS researchers are willing to share their data, but few are asked to do so, and in only a few domain areas do their funders or journals require them to deposit data. Few repositories exist to accept data in CENS research areas.. Data sharing tends to occur only through interpersonal exchanges. CENS researchers obtain data from repositories, and occasionally from registries and individuals, to provide context, calibration, or other forms of background for their studies. Neither CENS researchers nor those who request access to CENS data appear to use external data for primary research questions or for replication of studies. CENS researchers are willing to share data if they receive credit and retain first rights to publish their results. Practices of releasing, sharing, and reusing of data in CENS reaffirm the gift culture of scholarship, in which goods are bartered between trusted colleagues rather than treated as commodities.

## Introduction

With improvements in technology, tools, and communications, research data have become far easier to collect, save, manage, distribute, and reuse. Data-rich research environments can promote new fields of study, improve understanding of complex systems such as the Earth's climate, and lead to new products such as pharmaceutical drugs [1]. Accordingly, funding agencies, policy makers, research institutions, and journals are encouraging researchers to release their data. Data management plans, deposit requirements, linking of journal articles to the data reported in them, and development of community-specific data sharing policies all are part of this trend.

Policies that require or encourage the release of data are predicated on the assumption that those data are useful to others [2]. However, little is known about how researchers manage their data, or about when, how, or whether researchers will share their data. Even less is known about when, how, and whether researchers use data they have not collected themselves.

Challenges for leveraging research data are many, including disparate practices of individual scientists, teams, and research specialties; labor and expertise needed to manage data; lack of incentives to release data; variant intellectual property regimes; competing policies for data release and control; and the difficulty of defining “data” in any given research endeavor [2]. The slow adoption of tools and services such as data repositories are indications that technology alone cannot change scientists' practices; other social and cultural factors must also encourage data sharing. Policies for data sharing should rest upon knowledge of how researchers share data and how researchers use data that have been shared with them.

In this article we explore data sharing practices among scientists and technology researchers in a National Science Foundation Science and Technology Center. The range of scientific and technical applications, and the size and diversity of the Center for Embedded Networked Sensing (CENS), make it an ideal site to address behavioral and policy questions surrounding data sharing. CENS, which began in August, 2002, and whose funding as an NSF Center ended in July, 2012, developed and studied networked sensing systems for critical scientific and social applications through collaborations between engineers, computer scientists, and domain scientists. We focus on the willingness of these researchers to share their data, their motivations to share data, conditions they place on sharing, means by which they share data, sources from which they obtain data, and purposes to which they put data obtained from sources outside their research teams. The breadth and depth of analysis, covering multiple disciplines over a period of ten years, using multiple methods, yields a rich set of insights about data sharing and reuse.

## Literature Review and Background

Data long have been the cornerstone of science. Hence, the ability to share, reuse, and combine data offers scientists a wealth of opportunities: reanalysis of evidence, verification of results, minimized duplication of effort, and accelerated innovation [3]–[8]. Particularly appealing to the “big sciences” (large, long-lived, highly instrumented projects [9]) is Jim Gray's “fourth paradigm,” a computational, data-intensive approach to science that constitutes a new set of methods beyond empiricism, theory, and simulation [10]. Digital data offer the potential for greater returns on investment, provided that data are properly managed and are shared among researchers [11], [12]. Data sharing can include attaching datasets to published articles, depositing datasets in repositories, posting data on a personal or laboratory website, or fulfilling requests from other researchers for data. Different methods for sharing may be more or less effective, and the degree of usefulness, trustworthiness, and value of the shared data will vary widely between researchers and disciplines. For the purposes of this article, any form of release of research data for use by others constitutes data sharing.

The motivations for sharing data are diverse and reflect the interests of many stakeholders, from individual researchers to funding agencies. Arguments for sharing data include providing the ability to reproduce and verify the results of past research [13], [14], making the outputs of publicly funded research available to the public [15], allowing researchers to ask new questions of extant data [16], and advancing research and innovation [2], [10]. Research also suggests higher citation rates for papers whose associated data are publically available on the Internet [17]. Few standards yet exist for citing data, per se, though many efforts are under way to establish standards and practices for data citation and attribution [18], [19].

Despite these and other arguments that data have value in reuse, in only a few disciplines are scientists making their research data readily accessible to others on a consistent basis [20]. A recent survey in *Science* (2011) found that among their peer-reviewers, only 7.6% archive data in a community repository, while 88.7% archive data in university servers or laboratory computers, out of the immediate reach of other scientists [21]. Another study reported that only an estimated 1% of ecological data are accessible after the results have been published [8]. Partly in response to this low rate of data availability, a group of ecological sciences journals recently announced a joint policy for data release [22]. While a promising development, a 2009 study found that explicit journal policies requiring authors of journal articles to share data do not necessarily result in authors making their data available to outside researchers [23].

Tenopir et al, [24] studied general trends in data sharing by conducting an online survey of scientists. At the core of their sample population were members of the Data Observation Network for Earth (DataONE) consortium, which is a DataNet project funded by the National Science Foundation [25]. They sent the survey link to the project's members, to federal agencies that manage and produce large amounts of data, and to scientific researchers at universities, asking recipients to answer the survey and to distribute the link to others who might be interested. In total, 1329 scientists from North America, Europe, and Asia responded to the online survey, which Tenopir et al. estimate to be a 9% response rate from this snowball sample.

The small proportion of those contacted who chose to respond to the Tenopir et al. survey were strongly in favor of sharing data. Of the 1329 survey respondents, 75% agreed that they share their data with others, and 78% said they were willing to put at least some of their data in a central data repository with no restrictions. Respondents indicated strong interest in using datasets from other researchers, if the data were easy to access. Despite the overwhelming support for data sharing and reuse among respondents, only 46% made their data available on the Internet, 36% agreed that their own data are easy to access, and less than 6% made all of their data available. These results raise important questions about the complexities of data sharing and reuse but shed little light on the types and circumstances of data sharing or reuse. Surveys can reach far larger populations than can interview studies or ethnographies, but their validity is limited by low response rates and by the inability to observe actual behavior.

Much more is known about why researchers do *not* share data than about why they do share. Among the many reasons for not making data available are a lack of appropriate infrastructure [26], concerns about protecting the researcher's right to publish their results first [27], incentive systems that favor publishing articles over publishing data [28], difficulty in establishing trust in others' data [29], and the individual investment needed to preserve and manage data in ways that will be understandable and useful to others [30]. This is not to suggest that researchers are selfish, lazy, or greedy. Rather, these findings suggest that despite the current interest in managing, sharing, and reusing research data, the infrastructure and incentives to do so do not yet exist.

Another explanation for the lack of sharing is the “gift culture” of scholarship [31]–[34], [27]. Researchers exchange data, documents, specimens, and other intellectual resources with each other through trusted relationships. Data often are closely held, as they can be bartered for other data or resources [27]. If openly deposited for anyone to use, researchers may lose the ability to barter data privately, thus creating a disincentive for deposit [28]. The sharp difference in sharing rates reported in the Tenopir et al. survey (75% saying they share, 36% saying their data are easy to access, and 6% saying they make all their data available) similarly suggests that much sharing is private, rather than public.

Underlying the arguments for data sharing are assumptions that available data will be used or reused by others. However, surprisingly few studies have addressed how and when researchers reuse data they obtain from other researchers. Mayernik's [35] study found that CENS researchers could not readily imagine what uses others might make of their data, and so creating documentation to facilitate data reuse in the future was a low priority. Zimmerman's [36] study of ecologists defines reuse as “the use of data collected for one purpose to study a new problem” (p. 634). In ecology, reuse of data is complicated by the wide variety of variables collected in subtly different ways by different researchers. Faniel and Jacobsen [37] studied how and when earthquake engineering researchers reused data from other studies. These researchers relied heavily on their own domain expertise to assess the veracity and appropriateness of others' data. Among the most important factors were the quality of documentation, the ability to interpret the data, and the applicability to the problem at hand. Hartswood et al. [38], studying the reuse of mammography images, found that the data were very difficult to interpret once separated from contextual information about the patient. Such complexities and circumstantial matters in the creation and use of data are well known in the social studies of science, but only recently are being applied to the study of data practices and data sharing [28], [39], [40].

Researchers are under increased pressure to release data, whether by requirements of funding agencies or by journals in which they publish. As long as data continue to be treated as a supplement to the written record of science, little motivation exists for scientists to alter their behavior, and ultimately, efforts to mandate data sharing will fall short [12]. As a recent *Science* editorial put it, “We must all accept that science is data and that data are science, and thus provide for, and justify the need for the support, of much-improved data curation” [4]. A collaborative effort is needed to address data sharing and data reuse, one that supports the needs of scientists, researchers, funding agencies, and the public.

### Characteristics of Data

An agreed scope of what is meant by “data” is crucial for a discussion of data sharing. What researchers consider to be their data will influence whether they are willing to release those data. Influential factors include the methods by which data are collected, the forms of handling required, the availability of standard tools for analysis, and the purposes to which the data are to be put [2].

Most formal definitions of data tend to reify the notion of “data” as fixed objects. A case in point is this widely cited definition, that data are “A reinterpretable representation of information in a formalized manner suitable for communication, interpretation, or processing. Examples of data include a sequence of bits, a table of numbers, the characters on a page, the recording of sounds made by a person speaking, or a moon rock specimen” [41].

As Bruno Latour [40] put it long ago, documents are malleable, mutable, and mobile. Data are even more malleable, mutable, and mobile than documents as they tend to exist in small units, are linked to many other related units, and are difficult to interpret without considerable documentation and context. Because data are so context dependent, they are difficult to transfer across research groups, sites, and disciplines [42], [43]. The mobility of data also appears to be a function of the type of research by which the data are generated and by which they might be used. Data from big science (large teams, long-term projects, extensive instrumentation) may be great in volume but usually are consistent in structure. As more people are involved, and as time periods to design research and instrumentation become longer, greater consistency is required in the resulting data. Big data from big science are intended for sharing among big teams [44]–[46]. Conversely, in “small science,” which is coming to be known as “the long tail” of science, individuals and small teams collect data for specific projects. These data tend to be small in volume, local in character, intended for use only by these teams, and are less likely to be structured in ways that allow data to be transferred easily between teams or individuals. While “big data” is getting the attention, small science and the long tail appear to constitute the major portion of scientific funding [45], [47]–[50]. Making data from the long tail discoverable and reusable is emerging as a major challenge 51–53

Data, for the purposes of this article, are the objects – digital or physical – that researchers consider to be their sources of evidence for a given study. For the CENS researchers, data include sensor readings, temperature measurements, samples of water from lakes, streams, rivers, oceans, or beaches, software code, and diagnostics from physical hardware. Some researchers include as data laboratory and field notebooks, models, and figures and tables in publications. Data also are transformed through multiple states, from raw instrument readings, through cleaning, modeling, verification, replication, and other stages of analysis [54]–[59].

### Practices to Collect Data

The practices by which researchers collect data vary along many dimensions. Dimensions identified elsewhere include (1) the specificity of purposes for collecting data, ranging from exploratory research to building observatories, (2) scope of data collection, from describing particular events or phenomena to modeling systems, and (3) goal of the research, from empirical to theoretical [2]. Data collection practices differ also greatly between “big science” and the “long tail” of science. In big science areas such as astronomy and high energy physics, data collection tends to be well planned, well curated, highly visible, and collected by highly automated instrumentation. The majority of scientific data collection activity occurs in the long tail, however. In these cases, data are gathered via small projects that involve only one or a few researchers. The resulting datasets are specialized and not often preserved or reused [50].

### Sources of Data

Most scientific data are collected for scientific purposes. In contrast, the social sciences and humanities often draw upon data that were collected for non-research purposes, such as records of business and government, social activity, genealogy, or literary works [28]. Scientists collect much of their own data in laboratories and in the field, whether collecting observations, conducting experiments, or building models or simulations [7].

Many external sources of scientific data are available for use in investigations or for corroboration. Observatories are important sources of data on distributions of natural phenomena. These are institutions that systematically collect observations such as air quality, wind speed and direction, water quality, plant and animal species, soil, carbon flux, and astronomical objects. Examples include NEON and LTER in ecology [60]–[62], GEON in the earth sciences [63], [64], and synoptic sky surveys in astronomy such as the Sloan Digital Sky Survey, the Panoramic Survey Telescope & Rapid Response System, and the Large Synoptic Survey Telescope [65]–[67]. The most comprehensive observatories attempt to provide an integrated view of some whole entity or system, such as the earth or sky. Their value lies in systematically capturing the same set of observations over long periods of time. Global climate modeling, for example, depends upon consistent data collection of climate phenomena around the world at agreed-upon times, locations, and variables [68]. Astronomical and environmental observatories are massive investments, intended to serve a large community. Investigators and others can mine the data to ask their own questions or to identify bases for comparison with data from other sources.

Scientists also can obtain data from other kinds of repositories that aggregate data of a common type, but do not represent systematic capture of phenomena. GenBank, for example, is a repository of genetic data generated by individual researchers or research groups working on projects with narrow scope [69]. Repositories of social surveys, such as the Inter-University Consortium for Political and Social Research [70] gather important social surveys, but each survey may have its own data structure and codebook. Results of individual surveys are not easily combined. Software code repositories such as SourceForge, Free(Code) (formally Fresh Meat), and CodePlex [71]–[73] serve a function similar to repositories of survey data. Open source software can be deposited; others may reuse and improve upon the code. Software in these repositories may be described with basic metadata, but is not necessarily described consistently nor is any software package necessarily interoperable with other code in the repository.

Another useful, but less common source of data are registries. These are catalogs of datasets that allow researchers to indicate the existence of data without going through the process of adding their data to a repository [74]–[76]. Not all domains have data repositories where researchers could deposit their data, and not all researchers can or want to expose their data; in these cases a registry can still make the data visible for discovery. A registry entry may include a link to obtain data directly, or it may provide contact information and conditions for access to the data.

### Center for Embedded Networked Sensing

The research reported here was conducted at the Center for Embedded Networked Sensing (CENS), a National Science Foundation Science and Technology Center funded from 2002 to 2012. The Center supports multi-disciplinary collaborations among faculty, staff, and students of the five partner universities (UCLA, USC, Caltech, UC-Merced, and UC-Riverside), with research on developing and implementing innovative wireless systems. A typical research scenario is one in which scientists and technology researchers jointly develop and deploy a wireless sensor network in an environment that a scientific team wishes to observe. Both the sensor network and the environment are studied – the sensor for its effectiveness and ability to collect accurate data, and the environment for trends and patterns that can be found in the data collected by the sensors. Two of the authors of this paper have studied the data practices of researchers at CENS since the Center's inception, reporting elsewhere on aspects of data, collaboration, and scientific research [54], [55], [77], [59], [57], [58].

The data collected or used by CENS researchers in science and technology span the dimensions of specificity, scope, and goal that were identified in the previous section. CENS data are diverse and small in volume, and hence fall into the long tail of science. Much of the scientific research with embedded network sensing is exploratory. Teams go into the field with research questions about particular phenomena and may return to the laboratory either to test or to generate hypotheses. Some researchers model systems, and others use models of phenomena to design their data collection methods. Most of the scientific and engineering research is empirical, some of which leads to theoretical models of system and network behavior. CENS researchers are not in the business of building observatories, except on a very small scale. One of the participating sites is part of the University of California Natural Reserve system. James Reserve monitors local environmental conditions and streams them to a public website for anyone to use [78], [74].

**Figure 1:**
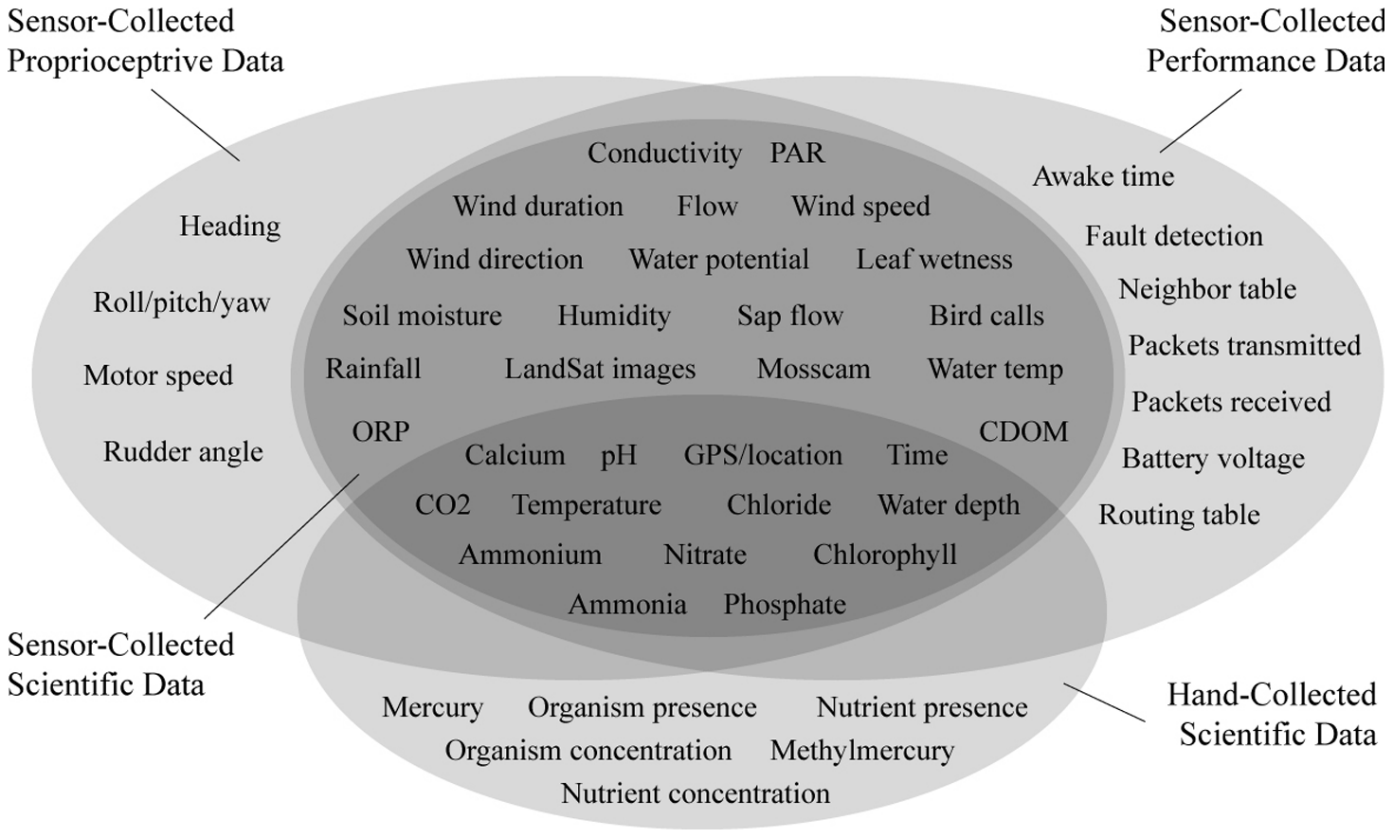
CENS data types organized by collection method and use (adapted from [47]).

The diversity of data created by CENS researchers is one of the reasons why it is such a productive site for studying data practices. Definitions of data differ from person to person and situation to situation [54]. Data collected during CENS deployments typically fall into four categories [59]: sensor-collected proprioceptive data, sensor-collected performance data, hand-collected application data, and sensor-collected application data. Each of the four data categories has multiple variables, as shown in Figure 1. The variables listed are only a subset of the overall inventory of CENS variables and data types.

Most CENS researchers are interested in the sensor-collected application data (center set in Figure 1), albeit for different purposes. Scientists are interested in discovering trends and patterns within numerical data such as growth patterns of plants in a particular lake. For them, the sensor-collected application data are evidence. The computer science and engineering researchers use the presence or absence of these data to monitor system and sensor performance. For them, these data are a means to test and improve their systems, software code, or tools [54].

For CENS participants, the data objects and their uses are deeply intertwined. For example, application scientists need to know the power levels of a barometric pressure sensor to be sure that the sensor was collecting accurate measurements, and technology researchers need the hand-collected samples to corroborate the sensor-collected measurements. Every participant has some form of “research data” associated with a field deployment or laboratory study. However, participants use other types of information to provide context for their research that were not identified as their “data.” We categorized these as foreground and background data, respectively [77]

“Foreground” data are the focus of the research, whether a field deployment or laboratory study. These forms of data are described as “core” or “primary” data, distinct from “background” data that serve other purposes. The sensor-collected application data in Figure 1 are foreground data to application scientists such as biologists and seismologists. Hand-collected application data (bottom set in Figure 1) tend to be foreground data only to application scientists. Sensor-collected performance data, such as packets transmitted, are foreground data to engineers studying network properties. To the roboticists, the proprioceptive data are foreground data.

Background data are those that are important to research activities but that are not necessarily reported in publications nor kept for future use or reuse. Most participants mention such types of data. The sensor-collected application data in Figure 1 can serve as background data to application scientists when used to verify the results of their hand-sampling, for example. The sensor-collected application data typically serve as background data to the engineers, who use them as system metrics, and to roboticists, who use them for navigation. The levels of quality required of data depend upon their intended use. Systems researchers use the presence or absence of sensor data as indicators of functionality and are not concerned with the values themselves. The application scientists, on the other hand, need precise values of observed phenomena.

### Research Questions

Although researchers are under increased pressure to manage and to share their data, the infrastructure to do so varies widely between the big science and the long tail. Big science fields have established community standards for data structures and associated repositories, tools, and services, whereas long tail science relies on local practices for data management, generic tools such as spreadsheets and statistical software, and have few options for contributing their data to repositories. CENS researchers epitomize the long tail of science. They are members of an NSF Science and Technology Center devoted to developing new technologies that will advance scientific research. Researchers in science, technology, and engineering collaborate to design new tools, new methods, and new interpretations of results. Each partner brings established methods and practices from his or her respective field. CENS is thus an idea locale to study data sharing and reuse.

We address the following research questions in this article:

1. What motivates researchers to share their data?
2. What conditions do researchers place on sharing their data?
3. How do researchers share data with others outside their research group?
4. What data are used that were not generated by a researcher's own group?
5. How are data from external sources used in research?

## Methods

Results presented here are based on interviews and ethnographic observation from 2002 to 2012 at multiple sites associated with the Center for Embedded Networked Sensing. We draw upon data from several waves of studies, selecting responses to questions about data sharing and situations where data sharing or reuse occur.

### Ethics Statement

The study method described below was submitted to and approved by the UCLA North Campus General Institutional Review Board. All participants interviewed and observed during the reported research provided their written informed consent, and were given the opportunity to withdraw from the study at any time. Interview and observational data collected as part of this research cannot be made publicly available because of conditions stipulated by the consent form. Specifically, that the data would not be made available without de-identifying the participants. This has been performed within the confines of the following publication, but is impossible with a full interview transcript where the participant has been asked to describe their research and career in some detail.

### Interviews

Interview data for this article are drawn from two rounds of data practices interviews, 43 in total, with participants from the CENS community. As CENS was founded in August, 2002, these interviews were conducted during the fourth (2005–2006) and eighth (2009–2010) academic years of the Center. These were optimal times to conduct interviews, as research activities were at their peak, collaborations were well established, and the technology was becoming more stable after a long startup period.

Interviews ranged from 45 minutes to 2 hours, with an average of 60 minutes per interview. For the first round of interviews, in 2005–2006, 22 participants were selected using stratified random sampling based on whether their research fell within the realm of technology or application science [79]. For the second round, in 2009–2010, 21 participants were selected using stratified random sampling based on the magnitude of their coefficient of betweenness centrality (i.e., the degree of connectedness they had with the rest of a co-authorship network constructed from CENS publications) [54], [80], [81]. Five persons were interviewed in both rounds of interviews due to the random draw. Although these interviews were not conducted as part of a proper panel study, the two rounds of interviews were conducted on a fairly stable population, and the interview questions were nearly identical. The two rounds of interviews are sufficiently comparable to identify changes in data sharing practices over time. However, we note that we interviewed more scientists (15) than technology researchers (7) in Round 1, and that the sample was more balanced between these groups (10 and 11, respectively) in Round 2, as shown in Table 1. By combining these two rounds of interviews with ten years of ethnographic observations, we can draw more comprehensive conclusions than by reporting the results separately.

**Table 1:** Interview participants and their distribution.

Research Area	Status	Round 1	Round 2	Totals
**Application Scientists**	Faculty	7	6	13
	Staff	5	2	7
	Student	3	2	5
**Technology Researchers**	Faculty	4	2	6
	Staff	1	2	3
	Student	2	7	9
**Totals**		22	21	43

At the time of both the first and second rounds of interviews, CENS was comprised of approximately 70 faculty and other researchers, about 140 student researchers, and several full-time research staff affiliated with the five participating universities. The CENS' roster fluctuated in size over the 10 years of the Center, peaking at about 300 members. The count also varied from year to year depending on the number of students, faculty, and staff associated with individual research grants affiliated with the Center at any given time [80], [81]. Interview passages note the year in which the interview took place, the job status of the interviewee (student, faculty, post-doc, or staff), and whether the person works in a scientific domain or in technology. The science domain participants are in the CENS areas of ecology, biology, marine sciences, seismology, and related areas. Environmental engineering participants are classified in the sciences as they are working primarily on science problems such as water quality and contaminant transfer. Technology participants include those in computer science and in other areas of engineering, primarily electrical engineering and robotics. Our research subjects are classified consistently as being science or technology participants in CENS in papers for which this distinction is relevant [54], [55]. Given the size of the sample, any more detail in disciplinary labels risks revealing the identity of our subjects. Other research on this population shows differences between those in the sciences and in technology, but finer distinctions in this population are not conclusive [54], [55].

Our interviews asked participants a series of questions about their data and data practices. Findings reported here are based on the following interview questions:

- Are you willing to share your data? Under what conditions?
- Do you ever put your data in a repository?
- Do you use data you did not generate yourself?

These open-ended questions drew rich responses. Where interviewees touched on these issues in response to other questions, those answers also were coded into the analysis reported here.

### Ethnographic Observation

Ethnographic research incorporated in this article includes field observations of participants performing research, laboratory and community meetings, and other events. As members of the CENS community, the authors interact with CENS researchers during formal gatherings, such as research reviews and retreats, weekly research seminars, and informal gatherings such as discussions within the lab and offices of CENS. Time spent in the field with researchers and participating in this community provides context for interpreting our interview results. We also include a brief case study of CENS' datasets from 2005 in the results. As members of the community, we were asked to review the usefulness of datasets posted to the CENS website.

### Qualitative Data Analysis

All interviews were audio recorded, transcribed, and complemented by the interviewers' memos on noteworthy topics and themes. Transcription of the interviews from Round 1 (2005–2006) totaled 312 pages, and the transcription of the Round 2 (2009–2010) interviews totaled 406 pages. Initial analysis of the transcripts and field notes revealed emergent themes. We developed a codebook and a full coding process using NVIVO software. Themes were tested and refined through further coding. Four coders (these three authors plus Matthew Mayernik, an author on other publications from this body of research) analyzed these data, with appropriate tests for inter-coder reliability.

## Results

The results are organized by research question. Findings from Round 1 and 2 interviews are reported separately and then compared in the discussion section.

### What motivates researchers to share their data?

CENS researchers – faculty, students, and staff alike – are willing to share their data. What data they are willing to share, when, with whom, and under what conditions, are much more difficult questions, with rich and nuanced answers.

#### Round 1

In Round 1, all of the 22 participants interviewed indicated that they were willing to share at least some of the data they had generated during their research with others outside their team. One reason for their willingness was the increase in the amount of data being generated. One participant noted the overabundance of data by the fourth year of CENS: “*There's tons and tons and tons of information out there now” (2006 Interview 2 – Faculty – Science)*.

This participant went on to explain how researchers need to share data so that others can pursue different lines of research later:

*The one criticism I think you will hear of observatories over the next 10 years is, well they did exactly what that one did. Why aren't they sharing their data? Why aren't they looking at these things together? Why are they making separate measurements of exactly the same thing, finding exactly the same result? And why are these guys doing it, when 10 years ago somebody did it over there and found the same thing? (2006 Interview 2 – Faculty – Science).*

Here the participant also identified the need to avoid repeating the work of the past by working together and sharing data, rather than by pursuing common lines of research independently.

Another participant collected data primarily to share them with others. Similar to the above participant, he recognized how the availability of data might positively affect the progress of science:

*I'm just motivated to see this type of data, microclimate data, really fine scale bird behavior data or whatever else we collect being used for scientific research in ways that people observing themselves can't do. I'm just motivated that I think it's going to allow biologists to ask a lot of questions and answer a lot of questions that before have just been completely impossible to do. So just kind of in general being able to ask more interesting scientific questions and being able to come up with better answers. That's my motivation as a biologist (2006 Interview 14 – Student – Science).*

For the above participant, the motivation for sharing was to enable other researchers to ask new questions, especially questions that would otherwise have been impossible to address.

Yet another participant noted that she shared data because she recognized that others may find the data interesting or useful. She explained that she posted some of her data online because, *“I think some of our data showing the changes, like the seasonal changes and patterns in the lake, would be of interest to other people”* (2006 Interview 10 – Staff – Science). Although she did not specify whom she thinks would be interested in her data, her realization that someone would want her data was motivation enough to try to make them available.

#### Round 2

In Round 2, all participants again indicated that they are willing to share some data, if not all, with others outside their research group.

One motivation was to make results of publicly funded research available to the public, as was noted by the following participant. Coincidentally, she was interviewed in both rounds and is the same person quoted immediately above in Round 1 (between the two rounds her status changed from staff to graduate student):

*For scientists, you don't really want to put all of your data on the web. But if it is funded by public money you know, for us we are dealing with things that are relevant to people and beaches. We want to share that (2009 Interview 3 – Student – Science).*

Some perceived direct advantages for sharing data. This participant, who develops open source robotic platforms, suggested that his research group would benefit from the adoption of their data due to network effects:

*If such a request arrives, then we immediately grant it because we would like to actually share all of our information and all of our systems to the wider academic community. And so such a request arrived before, and [we] provided the data immediately, and we not only share the data but also the platforms we developed. We would like other researchers to adopt them and use them in their research as well and there are many cases when we did that as well (2009 Interview 10 – Staff – Technology).*

### What conditions do researchers place on sharing their data?

Although the participants interviewed were willing, in principle, to share their data, they identified a number of conditions to be met before they would share. The most commonly cited conditions for sharing data were (1) first rights to publish results, (2) proper attribution to the data source, (3) familiarity between sharer and recipient, (4) funding agency expectations, and (5) the amount of effort required to share. Table 2 lists the conditions identified by the participants and the number of participants in each round who cited the condition in their interview. In Round 1, 16 of the 22 participants discussed specific conditions for sharing. These 16 participants identified 15 conditions; these conditions were mentioned a total of 36 times. In Round 2, 16 of the 21 participants discussed conditions. The latter 16 participants identified 13 conditions, which were mentioned a total of 36 times. Many participants listed multiple conditions for sharing their data; one participant in Round 2 named five different conditions. The only singular condition mentioned was retaining first rights to publish, which was the greatest concern overall.

**Table 2:** Conditions for data sharing.

“I will share my data if….”	Round 1	Round 2	Total
***Number of participants in Interview round***	***22***	***21***	***43***
***Number of participants who mentioned conditions***	***16***	***16***	***32***
I have first rights to publish the results from the data	15	5	20
I will receive proper attribution as the data source	5	2	7
The requestor is known to me or my group	2	4	6
My research funder expects me to share	2	4	6
Minimal effort is required to share	1	4	5
Sharing was negotiated in advance of exchange	1	3	4
The data are appropriately sized (not too big or too small)	1	3	4
Research and/or data are developed and stable		3	3
My community expects me to do so		3	3
Minimal effort was required to collect data	2		2
The data will be easily understood by others	1	1	2
The journal requires that the data be shared	1	1	2
Permission was granted by the PI on the project		2	2
Standard methods exist for interoperability	1		1
Shared data are not focus of participant's research	1		1
Data collection is part of my job description	1		1
I do not plan to commercialize the data or technology	1		1
Shared data will be re-shared with others	1		1
Data recipient and I address same research question		1	1
**Total Number of Mentions**	**36**	**36**	**72**

#### Round 1

In Round 1, most of the 16 conditions for sharing data were mentioned by only one or two participants, as seen in Table 2. Two conditions for sharing dominated: retaining first rights to publish results and attribution for the source of the data.

15 of 22 participants said that they would share data only in situations where the originating team retained the first rights to publish results from the data. This condition was by far the most frequently mentioned. As the participant below explains, researchers should only release their data when they are comfortable that the data are fully exploited in their own publications:

*I would feel robbed if I turned around and I looked at a paper and someone else published my work that I was planning on doing. Or even if someone did something that made what I was doing null or no longer interesting… I think what is more logical is that you collect data and you retain it for awhile until you get what you need out of it or at least until you are far enough along that someone can't come and usurp you (2006 Interview 1 – Student – Science).*

Five of the 22 participants in Round 1 were concerned about receiving proper attribution for the work they had done to collect and to share their data. As the researcher below explained, a standard for systematically citing data is necessary to encourage data sharing, especially for repositories that mediate the interaction between the person using the data and the person or team sharing the data:

*You would have to have, I think, standards for citation and for encoding the authorship of that data even if it hadn't necessarily been officially published. So I think some shared resources would be great, but I think people would be a little scared of dumping all their data into a repository, if there was a chance that someone would be able to just take that data and not cite it responsibly (2006 Interview 10 – Staff – Science).*

#### Round 2

In the second round of interviews, 13 conditions for sharing emerged, four of which were mentioned by at least four participants, as shown in Table 2. Concerns for attribution dropped to two mentions. Competition for publishing rights remained in first place, but without the clear margin in Round 1.

Five of the 21 participants stated that they would only share data on the condition that the originating team retained first rights to publish. The team or individual receiving the data was welcome to publish their own interpretations of those data thereafter.

Four participants from Round 2 would share data only with someone they knew. These concerns intersected with those of first rights to publish. One participant who mentioned both of these conditions said, *“If someone else called me that I didn't know, I would definitely think about… giving over data, because it makes it less publishable in even what we do”* (2009 Interview 7 – Faculty – Science). Another participant was willing to share data with almost anyone, but when she knows someone personally, she would invest extra effort to prepare and to share the data:

*I either refer people, if I don't know them at all, I refer them to the [Domain Data Repository]. So I am not handing over a lot of data. If I had worked very closely with the person in the past, then I might go out of my way to actually prep the data that they want and put on some site where they can easily download it (2009 Interview 13 – Faculty – Science).*

In Round 2, four of the 21 participants placed conditions on data sharing depending on their source of funding and the expectations of the funding agency. The following participant follows funding agency rules for when, whether, and how to release data for use by others:

*The Mexico data might not go into [X repository], because we have funding from the [private foundation] rather than from NSF. NSF would require that we deposit the data in [X repository]. But because we don't have NSF funding, we have [foundation] funding, depositing data in [X repository] would be out of the goodness of our hearts (2009 Interview 15 – Faculty – Science).*

In cases such as these, the researcher may be willing to share if contacted by another researcher, but if the funding agency does not require data to be put into a repository, the data may be maintained locally and not made readily accessible to others.

Four of 21 participants in Round 2 expressed concern about the amount of effort required to help someone else understand the data he or she might share. As this participant notes, he is more likely to share his data when he feels that the person receiving the data will not need much help or explanation to use them:

*That would be the main one, how much work would the sharing take. I would hope they would be collaborative, not take it and run. But I also would be, I also do know that some of the data sets are pretty complicated and the nuances of, it's not… I think it will be quite easy when we have the stations, because here is the station, there is a picture of it in the river, and dwell time series. And that would be much more shareable than, for example, [technology project] data set were, first of all you got to explain to him what [technology project] is, and then you gotta, okay and this is where it is in the river and its right here at this bend and there was some plants over here, and there was a log over here (2009 Interview 21 – Faculty – Science).*

### How do researchers share data with others outside their research group?

Our interview questions did not ask specifically what methods were used to share data. Other questions such as, “Are you willing to share your data?, Under what conditions?, and Do you ever put your data in a repository?” elicited a number of methods by which they share, as shown in Table 3. In Round 1, 21 of the 22 participants discussed methods they used to share data, for a total of 34 mentions. In Round 2, 15 of the 22 participants discussed methods for sharing data, also for a total of 34 mentions. Some participants mentioned more than one method that they had used to share data with others.

**Table 3:** Methods for sharing data.

Methods for Sharing Data	Round 1	Round 2	Total
***Number of participants interviewed***	***22***	***21***	***43***
***Number of participants mentioning methods to share data***	***21***	***15***	***36***
Fulfill personal requests	10	12	22
Post data to a website	15	6	21
Submit data to a repository	2	10	12
Data Publication	2	4	6
Supplement to published journal article	2	1	3
Submit data description to a registry	3	1	4
**Total Number of Mentions**	**34**	**34**	**68**

We identified subtle but important differences between the use of repositories and registries to share and to obtain data. They are coded as separate categories for sharing and for sources of data. Repositories contain data, whether from observatories or contributed by individual investigators. Registries record the existence of a dataset and include a brief description and contact information, but do not ingest the data, per se. Below we discuss the three methods most commonly mentioned for sharing data, whose order varies between interview rounds: (1) fulfill personal requests, (2) post data to a website, and (3) submit data to a repository. Also reported in this section is a complementary case study of datasets posted to the CENS website.

#### Round 1

In Round 1, the method of sharing mentioned by the most participants, 15 of the 22, was to share data by posting them on a local website. Five participants specifically used the CENS website to host data. This method allows anyone interested to discover and to download data without mediation by the researcher, as the following participant indicates:

*We have now an almost continuous long-term data set of that information. And we just put it in spreadsheet format and post it to our Web site and people can download it. And we tell them how to attribute it to the people who collected it (2006 Interview 12 – Faculty –Science).*

The data to which the above quote refers are ornithology observations collected in the California National Reserve associated with CENS. All use of the Reserve is tracked and reported to the funding agency, including uses of data collected there. Any project that wishes to conduct research at the Reserve, including CENS participants, must complete an application form. That form requires researchers to agree to the data sharing policy that allows these data to be posted.

In an effort to promote the release of CENS data, a website page was added to the CENS site in 2005 – concurrent with the Round 1 interviews – for members to post their datasets. Only seven datasets were posted, of varying types and formats. Our team was asked to evaluate the datasets and their usefulness. The seven datasets consisted of one simulation, three spreadsheets that contained time-series data in a comma-delimited format, source code for a tool, and one database where users could generate a query specifying sensors and time periods to create simple data visualizations or select the data for download in a CSV file. Each of the seven datasets contained a link for further information or access to a larger set of data. These links varied greatly in their destinations and in their stability. The most useful links were the three that led to a project landing page that described the data resources available for downloading and provided guidance for proper attribution. The other four links were of questionable value. One link led to a project description only, with no available data. Another link led to a set of data download instructions. However, that link pointed to a server that ceased to function within two weeks of posting the instructions. A third link led to a mislabeled page with a series of links that initiated download of various datasets, but without descriptions of those datasets. The fourth link initiated a download of a large data file with no warning. No other datasets were ever added to this page, and it eventually disappeared from the CENS website in 2009. In sum, we found that posting data or links to data, while well intentioned, did not necessarily result in making useful or usable data available.

In Round 1, ten of the 22 interviewees indicated that they had been contacted with a request to share data from a CENS project. Nine of these participants subsequently fulfilled the request; the tenth said he intended to comply but had not yet done so.

One participant responded to a data sharing request thusly:

*I can only think of one instance right now, but there's another team, or another scientist doing [phenomenon] studies in New Mexico, who wants to see how she's interpreting what she gets, and whether it's equivalent in any way to how we're interpreting what we get. So are we quantifying things in similar ways or not. So I'm trying to gather a subset of what we have, to send her, that she can then look at and compare with what she's collecting. It's like that quality control verification thing, to see if it matches (2006 Interview 21 – Staff –Science).*

The above data transfer required multiple actions. First, a data seeker identified the CENS participant as a potential data source. Second, the data seeker contacted the CENS scientific team. Third, the data seeker and CENS team member discussed what data existed, what use of the data was desired, and what data could identified and processed at CENS to be useful. Fourth, the CENS team member gathered and processed appropriate datasets. Fifth, these custom datasets were conveyed to the requestor.

While participants often mentioned that public repositories are a good method of sharing, only four participants in Round 1 could name a repository that they would use, and only two had deposited their data into a public, discipline-specific repository. The data being deposited, in both cases, were genetic in nature. Genetics is an area where data deposit is expected and where public repositories exist, as illustrated by this quote:

*We do this with our DNA data, if we publish an article then if there is a repository, a national repository like there is for DNA data, Gen Bank, we actually will put that information into Gen Bank (2006 Interview 2 – Faculty – Science).*

Ten of the 22 participants were unaware of repositories that would accept data from their type of research. A typical response when asked if they deposited their data into a repository is: *“Yeah. I mean, I know nothing about that. There's data repositories?”* (2006 Interview 1 – Student – Science).

#### Round 2

Participants from the Round 2 interviews also identified many different methods for data sharing. The same three methods were common, but repository deposit increased and posting data on websites decreased.

In Round 2, twelve of 21 participants had been contacted by someone outside their research group with a request to ask for data, and all twelve said they fulfilled these requests. This was the most common form of data sharing in Round 2.

One participant, when asked about being contacted to share data, explained that although he was generally willing to comply with requests, he tried to get a sense of who was asking, to gain more control over the release of his data:

*I generally try to give, I try to comply. I do weigh it a little bit because sometimes you can tell who, you know, can sort of handle it, and who is going to just be a lot of work, kind of keep coming back with questions. But I mean, we kind of have to give it to them. So I usually start off by sending them, you know, they can join our site now, give a quick little, a couple of lines like “Here's the site … these are where other files are. And if you are looking for some of the results then something here (2009 Interview 21 – Faculty – Science).*

In Round 2, only six of 21 participants said they make their data available on a website, which is a steep drop from 15 of 22 in Round 1. A common reason for posting was to refer requestors to the online source rather than to address data requests individually: *“In most cases the student ends up putting that on a website somewhere and sending people the link”* (2009 Interview 16 – Faculty –Technology).

By Round 2, ten of 21 participants had deposited data into a public, discipline-specific repository as can be seen in table 4. This is a steep increase from Round 1, in which we found little awareness of repositories. As with the Round 1 data, one of the participants deposited data in genomic repositories, such as GenBank. One of the participants mentioned contributing data to a CENS-built repository, SensorBase. Two participants from ecology mentioned contributing invasive species data to a larger invasive species mapping initiative. Four of these participants were computer scientists who had shared code or system details in a repository. For example, the following participant would deposit his data into the repository Free(Code) then known as “Fresh Meat:”

**Table 4:** Repositories used by participants to share data.

Name of repository	Round 1	Round 2	Total	Participant Discipline
***Number of participants interviewed***	***22***	***21***	***43***	
Anopheles Database	1	0	1	Marine Biology
Code.Google	0	1	1	Computer Science
Crawdad	0	1	1	Computer Science
EDDMaps	0	2	2	Ecology
Free(Code)	0	1	1	Computer Science
GenBank	1	1	2	Marine Biology
IRIS	0	2	2	Seismology
Personally managed SVN	0	1	1	Computer Science
SensorBase	0	1	1	Environmental Engineering
**Total Number of Mentions**	**2**	**10**	**12**

*…it will be locally hosted here at [my university] to Fresh Meat which is a big publication board for projects and just say hey, if you wanna look, check this out. …It's a repository of release announcements software. And you can search for different types of software (2009 Interview 17 – Student – Technology).*

Two participants working with seismology data deposit their data into seismology's community repository, IRIS, and one states, “We follow the national rules or the IRIS rules, where we make our data public in 2 years after the last instrument is out of the field” (2009 Interview 15 – Faculty –Science). This quote identifies several important factors for contributing data to a repository. First, a well-established disciplinary repository exists to accept their data. Second, the discipline has standards for describing and sharing data, which simplifies the process of depositing data. Finally, seismology has rules about what data must be deposited and when, which encourages (or requires) researchers to make their data available. These rules are subject to interpretation, of course. Researchers may delay removing the last piece of equipment from the field to gain additional time to analyze their data.

Eleven Round 2 participants did not contribute any of their data to a public, discipline-specific repository. As in the Round 1 interviews, some participants remained unaware of any relevant repositories in which they could deposit their data.

### What data are used that were not generated by a researcher's own group?

Findings for the fourth and fifth research questions are based on responses to the interview question, “Do you use data you did not generate yourself?” Participants identified sources of data originating outside of their research groups and then described their uses of them. Although in Round 1 interviews, 14 participants said that they use data they themselves did not generate, only nine participants mentioned specific data sources. Only one participant had contacted a researcher outside their group for data. In Round 2, of the 17 participants who answered that they use others' data, only 11 named specific data sources. Some participants named only one external data source, while others named two or three. Only three participants mentioned asking someone they did not know directly for data. Table 5 presents the observatories and repositories named by respondents, and the data they obtained from those sources.

**Table 5:** Where researchers find data for reuse and what data they use.

Name of Data Source	Observatory or Repository?	Data Used	Round 1	Round 2	Total
***Number of Participants in each Round***			***22***	***21***	***43***
***Participants Who Mentioned Data Sources***			***9***	***11***	***20***
CA Irrigation Management Information System	observatory	weather, solar radiation, soil temp		1	1
California Data Exchange Center (CDEC)	observatory	river conditions, river scales, gauging	1	1	2
Central and Northern CA Ocean Observing System (CeNCOOS)	Observatory/repository	unspecified		1	1
Crawdad	repository	802.11 measures		1	1
DARPA	observatory	photos	1		1
Free(Code)	repository	Software code		1	1
Heal the Bay	observatory	Malibu Watershed data, tidal charts	1		1
James Reserve (JR)	observatory/repository	Weather, environmental data, photos, web cam/Visitors' data	4	1	5
Macaulay Library at Cornell		Recordings of bird sounds	2		2
NASA	observatory	unspecified coastal ocean data		1	1
NASA's MODIS Satellite	observatory	spectral bands		1	1
NOAA	observatory	tidal height		1	1
NOAA's National Weather Service	observatory	point data	1		1
Satellite (unspecified)	observatory	images	1	1	2
Southern CA Coastal Ocean Observing System (SCOOS)	observatory/repository	unspecified coastal ocean data		1	1
TerraServer	observatory	remote sensing	1		1
UIUC Face Database	observatory	facial images	1		1
US Geologic Survey (USGS)	observatory	remote sensing, demographic data, gravitational data	1	1	2
**Total Number of Mentions**			**14**	**12**	**26**

#### Round 1

In Round 1, the 9 participants who mentioned using external data sources typically used types of data that included tidal charts, GIS data, or weather information; for example, “*We've been tapping into the James Reserves' weather sensor. They have that whole suite of meteorological instruments”* (2006 Interview 10 – Staff – Science).

The data most commonly mentioned were those collected by government observatories such as USGS, NASA, or the Southern California Coastal Ocean Observing System, or for-profit companies who collect data on a regular basis, as shown in Table 5. These observatories collect and disseminate digital data. When asked about using data that he had not created himself, one participant said, *“The only outside data that we routinely use is remote sensing and that comes from the USGS and from TerraServer”* (2006 Interview 12 – Faculty – Science). Data from USGS are free to researchers from the United States; imagery from the TerraServer must be purchased.

Observatories and repositories listed in Table 5 were the only external sources of data mentioned in Round 1 interviews. At that time (2005–06) none of those interviewed were reusing experimental, field, or laboratory data obtained from repositories or from other investigators.

#### Round 2

In Round 2, the 11 participants who mentioned external data sources commonly used climate data, tidal charts, GIS data, photos, or other similar types of data that they are able to obtain online, like the following participant: *“Most of the projects we're doing right now incorporate some kind of external map data or GIS data” (2009 Interview 6 – Faculty – Science).*

As in Round 1, most Round 2 participants obtained data from observatories like USGS, the California Data Exchange Center, or other regional sources such as these:

*I get some data from NASA. I get some data from local Web sites, the Southern California Ocean Observing System, (SCOOS), and also CeNCOOS, the Central and Northern California Coastal Observing System (2009 Interview 18 – Staff – Technology).*

The Southern CA Coastal Ocean Observing System, the Central and Northern CA Ocean Observing System, and James Reserve are classed as observatories because they collect and disseminate consistent measurements and also as repositories because they accept individual investigator's data from trusted sensors.

Only two participants in Round 2, both from technology, used data from other types of repositories. One used the Fresh Meat repository to find software code, and the other used the Crawdad repository, explained below:

*So, there's this website put together by some people at Dartmouth called Crawdad which has all these 802.11 or WiFi measurements. So there's like collection after collection after collection of data. I can just go and look at them and use it as they were (2009 Interview 2 – Student – Technology).*

### How are data from external sources used in research?

When researchers do obtain data from external sources, most use them for background or context purposes. Data from external sources rarely are the focal point, or foreground of research.

#### Round 1

The 14 participants (of 22) who said they use data they did not generate themselves typically do so for context or as baselines for their own research process. For example, one participant explained that before heading into the field, he refers to data about river conditions that are collected by the California Department of Water Resources and are distributed online through the California Digital Exchange Center:

*We use it every time when we're going down to the river to see what the conditions are and what they're projected to be, so we can know whether the banks are going to be under water or not, what the rough conditions are going to be (2006 Interview 13 and 2009 Interview 15 – Faculty – Science).*

Another participant purchased data to establish the baseline for the team's research. This team was developing a system to recognize specific bird sounds, so they needed recordings of these birds as training data for the system.

*We bought some recordings from Cornell from the same species that we're trying to recognize. And you know, just to perform preliminary tests (2006 Interview 16 – Staff –Science).*

Similarly, one participant uses data from the Heal the Bay project, “*when I'm trying to figure things out.”* Since the data are intended as context or background to the research, this participant explains that, *“I haven't used it in terms of my own analysis yet. The data are just as more of kind of a reference tool, I guess”* (2006 Interview 1 – Student – Science). Although he does not use the data for analysis, he suggests that he might blend this type of data with his own in the future.

#### Round 2

In Round 2, some participants used data for comparison, as described below:

*I would say we are using them for supplemental. I think the only time we are using it for comparison was when we did, where we were trying to use [our sensing system] to measure flow in the river and we did right next to the [USGS] gaging stations, we took a number there (2009 Interview 21 – Faculty – Science).*

In this case, the team uses external data as a supplement to their own data. They compare the measurements because the data were available, not because that was the purpose of the study.

Technology researchers also used data generated by others for comparison purposes. As in Round 1, the external data are used for background, rather than foreground, purposes:

*I've taken general published values for a lot of the drag coefficients and things like that. I haven't actually done model testing on our vehicle, so I'm making some approximations there from other people's testing and from the manufacturer of the vehicle (2009 Interview 18 – Staff – Technology).*

The use of external data to test algorithms was a common theme in Round 2 interviews. This computer scientist obtains network traces from others to test his algorithms:

*I definitely have for past projects. I mean I used like standard network traces gathered by different organizations to analyze what that network traffic looks like (2009 Interview 17 –Student – Technology).*

Another participant tests algorithms with photos obtained from a University of California Natural Reserve (and a CENS partner) or from other webcams that provide free photos: *“We used some data from the wired cameras that were deployed some years at James Reserve”* (2009 Interview 12 –Faculty – Technology). This participant uses images of birds to evaluate the ability of their algorithms to identify phenomena of interest from a steady stream of images.

## Discussion

Our two rounds of interviews with researchers, students, and staff in CENS, conducted in the fourth and eighth years of the Center, and complemented by ten years of ethnographic observation, reveal a rich picture of the interactions among types of data collected and shared, conditions for sharing data, methods by which data are shared, sources of external data, and uses of data from external sources. The discussion of results is organized by our five research questions.

### What motivates researchers to share their data?

Sharing data clearly is viewed as good behavior in science and technology research. All members of the CENS community, from both rounds of interviews, expressed willingness to share their data. However, our two rounds of interviews and long-term ethnography suggest that stated “willingness to share” may bear little relationship to actual release of data. Only about half of our participants could identify a case where they had been asked for their data, and few could identify a case in which they had asked other investigators for their data.

Thus, when it comes to sharing research data, actions speak louder than words. When asked in hypothetical terms whether they are willing to share their data, most researchers say they will share or that they do share [17], [24], [59], [56]. “Yes” is a predictable response for at least two reasons. One reason is that “willing to share” is now the pro-social answer. Funding agencies and journals are pressuring researchers to release their data. Few researchers are likely to make public pronouncements that they will not share their data in the face of this rising tide. The second reason is methodological. Social science research methodology discourages the use of hypothetical questions because the answers do not accurately predict what people actually will do if that situation occurs. More valid results are obtained by asking people about specific actions they have taken in specific situations, as we have done in this study.

CENS researchers identified cases where they did share data, and also explained their motivations for doing so. Motivations include facilitating other researchers' ability to pursue new lines of research, demonstrating the value of their own accomplishments, facilitating comparisons between methods and sites, and promulgating their technology as a basis for others' research. These motivations for sharing data align with policy arguments for sharing data, such as the ability to allow researchers to ask new questions and to advance research and innovation [2], [10], [16]. However, no cases were mentioned for which data were requested to replicate a study, an oft-cited purpose for sharing data [2], [13], [14].

Foreground data – those associated with the main research questions of a study – are most likely to be released, as background data often are not considered “data.” This finding further confirms and contextualizes results we report elsewhere [77]. Sharing occurs most often upon individual request, rather than via a data repository.

### What conditions do researchers place on sharing their data?

Although participants were willing, in principle, to share their data, they placed many conditions on data release. Conditions varied from researcher to researcher, regardless of research role or discipline. We identified 20 distinct conditions (Table 2), the most common of which was retaining first rights to publish results. This is an oft-stated concern in discussions of data sharing, and among the first to be identified [30], [27].

The concern for first rights to publish was more pronounced in the first round of interviews than the second, in which the conditions for sharing were more evenly distributed over 13 categories. Several explanations for this change are possible. One reason is simply that these samples are too small for a trend analysis. Another is that application scientists predominated in Round 1 (15 of 22 participants), and they appear to be more concerned about others claiming their findings than do technology researchers. Lastly, the sophistication of researchers regarding data sharing appeared to increase over the four years between interview rounds. By the time of the second round of interviews, embargoes and other mechanisms to assure that originating researchers maintain control over their data for reasonable periods of time were maturing.

In distant second place for data release was the condition that shared data be attributed properly to the originating researchers. The lack of standards for how to cite data and the lack of professional practice in citing data were concerns. Standards for data citation are now being developed and deployed, but these practices are far from mature [19].

Closely following in third place were two conditions. One is that the requestors are known and trusted individuals. Data sharing is viewed as a peer-to-peer relationship to many in the CENS community. This result is reinforced by our finding that fulfilling personal requests is the most common form of data release. If a researcher knows and trusts another researcher, he or she is more willing to release data, and even to do more work in preparing the data for release. Trust must be mutual, as the person sharing wants to ensure that the data will not be misused, and the person reusing the data needs to trust the accuracy and validity of data acquired. The necessity of trusting others' data, which in turn may depend on trust in others' data collection methods, is another consistent finding of studies on data sharing and collaboration [59], [37], [30], [36].

The other condition in third place is that investigators follow funding agency rules for data release. Sometimes this condition is stated generally, that data collected with public funds should be available to the public. At other times, the condition was stated in the negative, in that investigators felt less obligated to release data if the research were supported by a private foundation that did not expect sharing. This was a condition that researchers placed on themselves rather than on recipients of their data. Notably, these interviews were conducted prior to the National Science Foundation requirement for data management plans. NSF requirements for data sharing long predate the data management plans, however. Thus the CENS researchers' motivations to share are aligned with international public policy for data sharing [15].

### How do researchers share data with others outside their research group?

Among our most striking findings is the lack of use of repositories for sharing data. While CENS researchers do obtain data from repositories, as discussed further below, their use of repositories to deposit data ranks a distant third, after responding to individual requests and posting data on local websites.

Scientific data are contributed to repositories in the disciplines where those repositories exist, which is primarily for seismology and genomics, and code repositories for computer science. The rest of CENS data fall into the “long tail” of science and technology, which is the diverse array of datasets that have no obvious home [50]. Few CENS scientists could name a repository that would be a likely home for their data, nor could we find obvious repository homes that they had overlooked.

In both rounds of interviews, about half of the participants had been asked directly for data, and in both rounds they indicated that they fulfilled those requests. A close second in methods of sharing was to post data to a website, although this answer was much more common in Round 1 than in Round 2. Posting data on the website of the lab, university, or research center is sufficient to share them, in the eyes of many of our participants. This method enables researchers to respond to data sharing requests with a link to where datasets are posted. Thus website posting of data is often personal sharing.

Making data available and making data usable are not equivalent, however. Our case study of the datasets posted on the CENS site suggests that reuse is often difficult. The CENS site hosted a mix of data types and formats, with minimal documentation and few links to associated publications or other contextual materials. That site was taken down after about four years. Those interviewed who posted data did not mention plans for maintaining access to their data. Website posting thus lacks sustainability and may also lack discoverability, as datasets are not well indexed by search engines. In the Tenopir et al. [24] study, only 36% of respondents agree that their data are easy to access, although “easy access” is undefined. We did not ask specifically about perceived ease of access, but it is clear that most CENS data are not readily discoverable or usable. CENS data most often are identified through publications or through contact with the investigators. CENS teams sometimes will document data further upon request of trusted colleagues.

Seismology is a particularly interesting case of data release and sharing. Data from research conducted with U.S. federal funds must be deposited in IRIS – the community repository for seismic data – within two years after the last instrument is removed from the ground. In our small sample of seismologists, we found nuanced attention to these requirements. Removal of instruments may be delayed to gain more time for data analysis prior to release of the data. Seismic data resulting from private foundation funds may or may not be deposited in IRIS.

Another important case is the contribution of software code to repositories. Software code and models are among the most important research products of CENS, particularly in the view of researchers in computer science and engineering. Here the motivations to deposit are somewhat different than for other forms of scientific data. If enough other researchers reuse a team's software, then the software may become a common platform through network effects. The originating team gains community advantages by building related tools and by having other researchers build tools that interoperate with theirs. This is a common business strategy, and one more amenable to software than to sensor data.

### What data are used that were not generated by a researcher's own group?

CENS researchers do use data they do not collect themselves, mostly drawn from observatories and other public repositories. A total of 18 sources were mentioned, most of which were observatories, as shown in Table 4. These data were typically observations of phenomena or conditions in an area under study, such as weather patterns or wireless network calibrations. CENS technology researchers retrieve software from code repositories and also submit their own software to these repositories. A few participants mention asking other researchers directly for data. Given how little use CENS researchers make of other investigators' data, it is not surprising that they cannot readily imagine what uses others might make of their data, as Mayernik [35] found in another CENS study.

No one mentioned seeking data from other researchers' web sites. The apparent lack of reciprocity has two related explanations. One is that website posting is a convenient form of personal exchange. Rather than moving large files by email, ftp, Dropbox, or other means, researchers simply post datasets locally and send the requestor a link. The other explanation is that web posting of datasets is intended for exchange among collaborators and not for discovery. Our case study of datasets posted to the CENS websites confirms that data posted on local websites often are poorly documented and poorly maintained. Thus, they are difficult to discover, locate, or interpret.

### How are data from external sources used in research?

CENS researchers use external data, principally drawn from observatories, as baselines, context, calibration, or other forms of background for their research. Similarly, when others have asked CENS researchers for data, the form of request suggests that data were sought for background purposes. External sources of software are used to test algorithms; this also appears largely to be a background activity. In sum, the majority of participants in our studies reported using some data or software they did not generate themselves, and all of these uses appear to serve background roles.

No cases were mentioned, nor observed in the ethnographies, where data from external sources served foreground purposes for research. Possible explanations are that it is often more difficult to discover appropriate data, to trust data generated by others, or to use and interpret others' data [37], [30], [36], yet data from public observatories are especially important and trusted sources. Because the data from these observatories are not the foreground of the study, researchers may not view them as “using the data,” and may not cite them as sources in their papers [18]. Thus, in sensor network research, data are not being mined and integrated to ask new questions, as the Fourth Paradigm would predict [10]. The Fourth Paradigm notion is more apt for “big data” than for the long tail of science, however.

## Conclusions

Our ten years of studying science and technology research at the Center for Embedded Network Sensing – throughout the full life span of this National Science Foundation Science and Technology Center – yields a rich description of the data sharing and reuse practices of the researchers at CENS. We address the question posed in the article title, “if we share data, will anyone use them?” The answer varies by the characteristics of the data, the method of sharing, and the types of uses that might be made of the data. Data sharing in CENS, and presumably in other types of long tail science, is demand-driven rather than supply-driven. Data are not readily discoverable because investigators do not contribute them to repositories, accompanied by metadata and documentation. The effort to make data discoverable is difficult to justify, given the infrequency with which investigators are asked to release their data.

The lack of demand and the lack of discoverability appear to be a chicken-and-egg problem, which we divide into three sets of implications: practices for releasing and sharing data, practices for using external sources of data, and requirements for scholarly infrastructure to support the long tail of science and technology research.

### Releasing and Sharing Research Data

In the few domain areas of CENS where data release is required by funding agencies, principally seismology and genomics, investigators contribute their data to the appropriate repositories. Software code and models also are deposited in code repositories, following open source practices in these areas of computer science and engineering. For most scientific domain areas of CENS, however, suitable repositories do not exist to accept their data. This too is a chicken-and-egg problem endemic to the long tail of science and technology. Insufficient data are released to justify developing a repository, and insufficient demand exists to justify releasing and depositing data. As Mayernik [35] found, CENS investigators invest little effort in metadata creation because they cannot foresee who might use their data.

In most areas of CENS, data sharing is a personal matter. Investigators share data with colleagues they know and trust, and when asked to do so. This finding reaffirms the gift culture of scholarship [27], [31]–[34]. Researchers will trade valuable intellectual goods with each other. Data are not treated as commodities to be traded on an open market, at least not in the CENS type of long-tail research.

Most studies of data availability [8], [20], [21] focus on research data that are openly discoverable online. Our findings show that repository use and online searching are an incomplete view of data sharing. While CENS scientists do post some datasets on their website, they usually do so in support of personal requests. Posting a link to a spreadsheet, for example, is a form of data sharing. However, such links are not readily discoverable, nor are the contents of the files necessarily useful. It is the documentation and tacit knowledge gained through personal exchange that make these files valuable.

We also find that conditions for sharing matter. Investigators want credit for their data, both in terms of first rights to publish their findings and in attribution for any reuse of their data. Another concern that echoes other findings is that data not be misused or misinterpreted [28], [82]–[89]. When exchange is personal, it is easier to maintain a degree of control over the uses of a team's data.

CENS researchers are understandably concerned about the amount of effort required to make their data useful to others. They are more willing to invest effort in documenting data for people they know and trust, again reinforcing the personal nature and gift culture of data sharing. Issues of interpretability of borrowed data are especially acute in the long tail of science and technology. Data handling practices range from artisanal to industrial [2], [27], [90]. Most CENS data handling is artisanal, as often occurs in the long tail. These data are labor-intensive to collect, such as hand-gathered samples of water and soil, and to process, such as reconciling sensor time stamps with the hand-sampling. Some CENS data handling, in areas such as seismology, is further along the continuum toward industrial data collection. Once placed in the field, seismic sensors can capture data automatically and reliably for months at a time. Even in seismology, however, sensors can require considerable tending. Our partners in seismology often placed experimental instruments in less-than-ideal conditions, subject to damage by weather, animals, or vandals, and out of reach of cell towers and satellites for automatic retrieval of data. Interpreting such data requires extensive contextual knowledge of how they were collected and processed.

### Reusing Research Data

CENS researchers do reuse data collected by others. Their primary external sources of data are observatories, which collect consistent data on natural phenomena. Records of climate, flora, and fauna, all provide essential background data for comparison, calibration and “ground truthing” of CENS research [54]. CENS researchers sometimes obtain data from other teams, and that also appears to be for background uses. Similarly, it appears that most data borrowed from CENS was sought for background uses such as verification of instruments and design of field research.

CENS researchers appear to collect all of their own foreground data, whether physical samples or sensor readings. Foreground data are those that are the focus of research questions for a given study, whereas background data are those that provide context or calibration [77]. The same data can be foreground to one researcher and background to another, even on collaborating teams; the distinction is in the *use* of the data.

### Implications for scholarly infrastructure

Despite great pressure to share research data, and consistent findings that researchers are willing in principle to share their data, relatively little research data are shared or reused. Is this a failure of practice, of policy, or of science? Is it a failure to comprehend the nature of scholarship? Or does the data sharing imperative simply address the wrong problem?

Data sharing is perhaps better understood as the problem of making best use of research resources. Researchers produce large amounts of data, some of which may be useful to others. Making those data useful to others requires a substantial investment in documentation, and often in interpersonal negotiation, above and beyond the conduct of the research per se. It is not possible to justify making that level of investment in all data just in case someone, somewhere, at some future time, might wish to use them. The originating investigator bears the cost of data preparation. Other entities such as data repositories, universities, libraries, and funding agencies are likely to bear the cost of curating those data for sustainable access. Unknown – and often non-existent – reusers reap the benefits. This equation is not viable in economic or social terms.

Thus, the better question to ask is which data are worth the investment for reuse? This is yet another chicken-and-egg problem, unfortunately. Data for which demand exists should be kept and curated. Demand arises only if data are discoverable, accessible, and usable. How can demand be built until a critical mass of useful data are available? Who should bear the burden of building that critical mass?

The answers are many and complex, especially in the long tail of science and technology research. In the big sciences, the cost of instruments, data collection, and data curation can be amortized over large numbers of users. Projects such as the Sloan Digital Sky Survey have yielded manifold more papers, dissertations, and educational activities than the SDSS investigators alone could have been produced. Research on the human genome accelerated when gene sequences were aggregated in public repositories. These are big wins, with big data, at the industrial data production end of the spectrum.

Some CENS scientists are producing orders of magnitude more data than was possible with artisanal methods alone. Others are asking new questions made possible by the greater granularity of data collection offered by sensor networks, and improved scientific validity made possible by combining sensor and hand sampling data. They have scaled up their data collection, but it will never be on a par with astronomy or genomics. Far fewer people will be interested in the root growth of mini-rhizomes, wind patterns in the San Jacinto mountains, or biological triggers of harmful algal blooms. Their science, however, may be equally significant. The findings of CENS scientists and technology researchers may offer critical insights to climate change and to modeling of biological phenomena, for example. These data should not be lost, but neither can we expect these researchers to double their investment in each project just to assure that their data might be useful to some unknown future user. This is the classic long tail problem.

The technical, policy, and service infrastructure to support scholarly research must address the characteristics and needs of long tail science as well as the needs of big science [51]. Some of the infrastructure will serve all parties, such as high capacity data storage and transmission, workflow tools, and data visualization tools. Scientists in the long tail need better tools to collect and manage their data, especially at the early stages of data capture. Here the difficulty is to find tools that are adaptable to diverse local practices. What works for habitat ecology may not be useful to roboticists, but tools should not create silos of small communities. Attention to the social factors is essential. The value of private sharing between interested parties should be acknowledged, even celebrated. Data repositories fill important niches, but they are expensive to sustain and are not the only means to support data reuse and discovery. In prior work we have recommended the development of data registries which expose high-level metadata about datasets with a very low barrier to submission, but this approach has not been around long enough to provide concrete results [74].

Data creators deserve attribution and credit. Observatories and repositories also deserve credit for the roles they play. Our findings show that researchers may not cite those sources, thus usage is probably much greater than citation metrics suggest. As these mechanisms mature, and to the extent that they become embedded in reward systems, they will promote data reuse. The role of education should not be underestimated. While CENS researchers are on the leading edges of their respective fields, most lack expertise in data management or data curation. As the volume of data produced in the long tail of science accelerates, so will the demand for this expertise increase in graduate training.

Despite our efforts to assist CENS in building a uniform strategy for data management over the decade-long life of the Center, we found more differences than commonalities in the needs of CENS teams [54], [55], [57], [77]. The wide range of data sharing and reuse practices identified in CENS suggests the richness and variance that is likely to exist in other slices of the long tail of science and technology research. These researchers are not alone in needing better tools, services, and skills to manage their data. Infrastructure to facilitate the exploitation of those data must respect and honor the breadth of their research activities. Data sharing is not an end in itself, but rather a means to leverage knowledge for the advancement of science and technology.

## References

[1] Hey T, Trefethen AE (2005) Cyberinfrastructure for e-Science. Science 308: 817–821 doi:10.1126/science.1110410 15879209 10.1126/science.1110410

[2] Borgman CL (2012) The conundrum of sharing research data. Journal of the American Society for Information Science and Technology 63: 1059–1078 doi:10.1002/asi.22634

[3] Committee on Issues in the Transborder Flow of Scientific Data, National Research Council (1997) Bits of Power: Issues in Global Access to Scientific Data. National Academies Press. 250 p. Available: http://books.nap.edu/catalog.php?record_id=5504#toc. Accessed 2013 Apr 2.

[4] Hanson B, Sugden A, Alberts B (2011) Making Data Maximally Available. Science 331: 649–649 doi:10.1126/science.1203354 21310971 10.1126/science.1203354

[5] Kahn SD (2011) On the Future of Genomic Data. Science 331: 728–729 doi:10.1126/science.1197891 21311016 10.1126/science.1197891

[6] Lyon L (2007) Dealing with data: Roles, rights, responsibilities, and relationships. UKOLN. 65 p.

[7] National Science Board (U.S.) (2005) Long-Lived Digital Data Collections: Enabling Research and Education in the 21st Century. [Arlington VA]: National Science Foundation. 87 p. Available: http://www.nsf.gov/pubs/2005/nsb0540/nsb0540.pdf. Accessed 2012 Apr 2.

[8] Reichman OJ, Jones MB, Schildhauer MP (2011) Challenges and Opportunities of Open Data in Ecology. Science 331: 703–705 doi:10.1126/science.1197962 21311007 10.1126/science.1197962

[9] Price DJdS (1963) Little Science, Big Science. New York, NY, USA : Columbia University Press. 119 p.

[10] Hey T, Tansley S, Tolle K, editors(2009) The Fourth Paradigm: Data-Intensive Scientific Discovery. Redmond, Washington: Microsoft Research. 287 p. Available: http://research.microsoft.com/en-us/collaboration/fourthparadigm/contents.aspx. Accessed 2013 Apr 2.

[11] Berman F, Lavoie B, Ayris P, Cohen E, Courant P, et al.. (2010) Sustainable Economics for a Digital Planet: Ensuring Long-Term Access to Digital Information: Final Report of the Blue Ribbon Task Force on Sustainable Digital Preservation and Access. Available: http://brtf.sdsc.edu/biblio/BRTF_Final_Report.pdf. Accessed 2013 Apr 2.

[12] Buckland M (2011) Data management as bibliography. Bulletin of the American Society for Information Science and Technolgy 37: 34–37 Available: http://www.asis.org/Bulletin/Aug-11/AugSep11_Buckland.html. Accessed 2013 Apr 3.

[13] Jasny BR, Chin G, Chong L, Vignieri S (2011) Again, and Again, and Again …. Science 334: 1225 doi:10.1126/science.334.6060.1225 22144612 10.1126/science.334.6060.1225

[14] Stodden V (2009) The Legal Framework for Reproducible Scientific Research: Licensing and Copyright. Computing in Science and Engineering 11: 35–40 doi:10.1109/MCSE.2009.19

[15] Organisation for Economic Co-Operation and Development (2007) OECD Principles and Guidelines for Access To Research Data From Public Funding. Paris: Organisation for Economic Cooperation and Development. 24 p. Available: http://www.oecd.org/dataoecd/9/61/38500813.pdf. Accessed 2013 Apr 2.

[16] Whitlock MC (2011) Data archiving in ecology and evolution: best practices. Trends in Ecology & Evolution 26: 61–65 doi:10.1016/j.tree.2010.11.006 21159406 10.1016/j.tree.2010.11.006

[17] Piwowar HA, Day RS, Fridsma DB (2007) Sharing Detailed Research Data Is Associated with Increased Citation Rate. PLoS ONE 2: e308 doi:10.1371/journal.pone.0000308 17375194 10.1371/journal.pone.0000308 PMC1817752

[18] Borgman CL (2011) Why are the attribution and citation of scientific data important? Available: http://sites.nationalacademies.org/PGA/brdi/PGA_064019. Accessed 2013 Apr 2.

[19] National Academy of Sciences (2012) For Attribution – Developing Data Attribution and Citation Practices and Standards: Summary of an International Workshop. Available: http://www.nap.edu/openbook.php?record_id=13564. Accessed 2013 Apr 2.

[20] Nelson B (2009) Data sharing: Empty archives. Nature News 461: 160–163 doi:10.1038/461160a 10.1038/461160a 19741679

[21] Staff (2011) Challenges and Opportunities. Science 331: 692–693 doi:10.1126/science.331.6018.692 21311002 10.1126/science.331.6018.692

[22] Joint Data Archiving Policy (2011) Available: http://datadryad.org/jdap. Accessed 2013 Apr 2.

[23] Savage CJ, Vickers AJ (2009) Empirical Study of Data Sharing by Authors Publishing in PLoS Journals. PLoS ONE 4: e7078 doi:10.1371/journal.pone.0007078 19763261 10.1371/journal.pone.0007078 PMC2739314

[24] Tenopir C, Allard S, Douglass K, Aydinoglu AU, Wu L, et al (2011) Data Sharing by Scientists: Practices and Perceptions. PLoS ONE 6: e21101 doi:10.1371/journal.pone.0021101 21738610 10.1371/journal.pone.0021101 PMC3126798

[25] Data Observation Network for Earth (DataONE) (n.d.). Available: https://www.dataone.org/. Accessed 2013 Apr 2.

[26] Lynch C (2008) Big data: How do your data grow? Nature 455: 28–29 doi:10.1038/455028a 18769419 10.1038/455028a

[27] Hilgartner S, Brandt-Rauf SI (1994) Data access, ownership and control: Toward empirical studies of access practices. Science Communication 15: 355–372 doi:10.1177/107554709401500401

[28] Borgman CL (2007) Scholarship in the digital age: information, infrastructure, and the Internet. CambridgeMA : MIT Press. 360 p.

[29] Committee on Ensuring the Utility and Integrity of Research Data in a Digital Age, National Academy of Sciences (2009) Ensuring the Integrity, Accessibility, and Stewardship of Research Data in the Digital Age. Washington, D.C.: National Acadamies Press. 180 p. Available: http://www.nap.edu/catalog.php?record_id=12615. Accessed 2013 Apr 2.

[30] Feijen M (2011) What researchers want. Available: http://www.surffoundation.nl/en/publicaties/Pages/Whatresearcherswant.aspx. Accessed 2013 Apr 2.

[31] Lyman P (1996) What Is a Digital Library? Technical, Intellectual Property, and the Public Interest. Daedalus 125: 1–33 Available: http://www.jstor.org/stable/20027384. Accessed 2013 Apr 2.

[32] Merton RK (1994) Scientists' Competitive Behavior Is Not Peculiar To Our Competitive Age. The Scientist 8: 12–14 Available: http://www.the-scientist.com/?articles.view/articleNo/28538/title/Scientists-Competitive-Behavior-Is-Not-Peculiar-To-Our-Competitive-Age/. Accessed 2013 Apr 3.

[33] Mauss M (2006) The Gift: The Form and Reason for Exchange in Archaic Societies. Psychology Press. 228 p.

[34] Merton RK, Zuckerman H (1973) Institutionalized patterns of evaluation in science. In: Merton RK, editor. The Sociology of Science: Theoretical and Empirical Investigations. Chicago: University of Chicago Press. pp. 460–497.

[35] Mayernik MS (2011) Metadata Realities for Cyberinfrastructure: Data Authors as Metadata Creators. SSRN eLibrary. Available: http://papers.ssrn.com/sol3/papers.cfm?abstract_id=2042653. Accessed 2013 Apr 2.

[36] Zimmerman AS (2008) New Knowledge from Old Data: The Role of Standards in the Sharing and Reuse of Ecological Data. Science Technology Human Values 33: 631–652 doi:10.1177/0162243907306704

[37] Faniel IM, Jacobsen TE (2010) Reusing Scientific Data: How Earthquake Engineering Researchers Assess the Reusability of Colleagues' Data. Computer Supported Cooperative Work 19: 355–375 doi:10.1007/s10606-010-9117-8

[38] Hartswood M, Procter R, Taylor P, Blot L, Anderson S, et al.. (2012) Problems of data mobility and reuse in the provision of computer-based training for screening mammography. Proceedings of the 2012 annual Conference on Human Factors in Computing Systems: ACM Conference on Human Factors in Computing Systems (CHI). Austin, TX, USA: ACM Press. Available: https://www.escholar.manchester.ac.uk/uk-ac-man-scw:150474.

[39] Bowker GC (2005) Memory Practices in the Sciences. Cambridge, Mass.: MIT Press. 261 p.

[40] Latour B (1987) Science in Action: How to Follow Scientists and Engineers through Society. Cambridge, MA: Harvard University Press. 288 p.

[41] Consultative Committee for Space Data Systems (2002) Reference Model for an Open Archival Information System (OAIS). RECOMMENDATION FOR SPACE DATA SYSTEM STANDARDS. Available: http://public.ccsds.org/publications/RefModel.aspx. Accessed 2013 Apr 2.

[42] Gitelman L, editor (2013) “Raw Data” Is an Oxymoron. CambridgeMA : MIT Press. 192 p.

[43] Kanfer AG, Haythornthwaite C, Bruce BC, Bowker GC, Burbules NC, et al (2000) Modeling distributed knowledge processes in next generation multidisciplinary alliances. Information Systems Frontiers 2: 317–331 Available: http://www.ingentaconnect.com/content/klu/isfi/2000/00000002/F0020003/00277141. Accessed 2013 Apr 15.

[44] Aronova E, Baker KS, Oreskes N (2010) Big Science and Big Data in Biology: From the International Geophysical Year through the International Biological Program to the Long Term Ecological Research (LTER) Network, 1957–Present. Historical Studies in the Natural Sciences 40: 183–224 doi:10.1525/hsns.2010.40.2.183

[45] Birney E (2012) The making of ENCODE: Lessons for big-data projects. Nature 489: 49–51 doi:10.1038/489049a 22955613 10.1038/489049a

[46] Lynch C (2008) Big data: How do your data grow? Nature 455: 28–29 doi:10.1038/455028a 18769419 10.1038/455028a

[47] boyd danah (2012) Crawford K (2012) Critical Questions for Big Data. Information, Communication & Society 15: 662–679 doi:10.1080/1369118X.2012.678878

[48] Data standards urged (2012) Nature 492: 145–145 doi:10.1038/nj7427-145a

[49] Data's shameful neglect (2009) Nature 461: 145–145 doi:10.1038/461145a 10.1038/461145a 19741659

[50] Heidorn PB (2008) Shedding Light on the Dark Data in the Long Tail of Science. Library Trends 57: 280–299 doi:10.1353/lib.0.0036

[51] Foster I, Borgman CL, Heidorn PB, Howe W, Kesselman C (2013) Empowering Long Tail Research. Available: https://sites.google.com/site/ieltrconcept/ Accessed 2013 Apr 4.

[52] ICiS - Institute for Computing in Science: Big Data and Long Tails: Addressing the Cyber-Infrastructure Challenges for Research on a Budget (2012). Available: http://icis.anl.gov/programs/summer2012-3b. Accessed 2013 Apr 2.

[53] Stop Hyping Big Data and Start Paying Attention to “Long Data” | Wired Opinion (n.d.). Wired Opinion. Available: http://www.wired.com/opinion/2013/01/forget-big-data-think-long-data/. Accessed 2013 Apr 3.

[54] Borgman CL, Wallis JC, Mayernik MS (2012) Who's Got the Data? Interdependencies in Science and Technology Collaborations. Computer Supported Cooperative Work 21: 485–523 doi:10.1007/s10606-012-9169-z

[55] Mayernik MS, Wallis JC, Borgman CL (2013) Unearthing the infrastructure: Humans and sensors in field-based research. Computer Supported Cooperative Work 22: 65–101 doi:10.1007/s10606-012-9178-y

[56] Wallis JC, Borgman CL, Mayernik MS, Pepe A, Ramanathan N, et al.. (2007) Know Thy Sensor: Trust, Data Quality, and Data Integrity in Scientific Digital Libraries. Proceedings of the 11th European Conference on Research and Advanced Technology for Digital Libraries. Budapest, Hungary: Berlin: Springer, Vol. 4675. pp. 380–391. doi:10.1007/978-3-540-74851-9_32

[57] Wallis JC, Borgman CL, Mayernik MS, Pepe A (2008) Moving archival practices upstream: An exploration of the life cycle of ecological sensing data in collaborative field research. International Journal of Digital Curation 3: 114–126 doi:10.2218/ijdc.v3i1.46

[58] Borgman CL, Wallis JC, Mayernik MS, Pepe A (2007) Drowning in data: Digital library architecture to support scientific use of embedded sensor networks. Joint Conference on Digital Libraries, Association for Computing Machinery. Vancouver, British Columbia, Canada: ACM Press. pp. 269–277. doi:10.1145/1255175.1255228

[59] Borgman CL, Wallis JC, Enyedy N (2007) Little Science confronts the data deluge: Habitat ecology, embedded sensor networks, and digital libraries. Int J Digit Libr 7: 17–30 doi:10.1007/s00799-007-0022-9

[60] National Ecological Obsevatory Network (n.d.). Available: http://www.neoninc.org/. Accessed 2013 Apr 2.

[61] Porter JH (2010) A Brief History of Data Sharing in the U.S. Long Term Ecological Research Network. Bulletin of the Ecological Society of America 91: 14–20 doi:10.1890/0012-9623-91.1.14

[62] U.S. Long Term Ecological Research Network (2012). Available: http://www.lternet.edu/. Accessed 2013 Apr 2.

[63] GEON - Geosciences Network (n.d.). Available: http://www.geongrid.org/ Accessed 2013 Apr 2.

[64] Ribes D, Bowker GC (2008) Organizing for multidisciplinary collaboration: The case of the Geosciences Network. In: Olson GM, Zimmerman A, Bos N, editors. Science on the Internet. Cambridge, MA : MIT Press. pp. 311–330.

[65] Pan-Starrs - Panoramic Survey Telescope & Rapid Response System - Institute for Astronomy, University of Hawaii (n.d.). Available: http://pan-starrs.ifa.hawaii.edu/public/. Accessed 2013 Apr 2.

[66] Sloan Digital Sky Survey (2011). Available: http://www.sdss.org/. Accessed 2013 Apre 2.

[67] The New Sky | Large Synoptic Survey Telescope (n.d.). Available: http://www.lsst.org/lsst/. Accessed 2013 Apr 2.

[68] Edwards PN (2010) A Vast Machine: Computer Models, Climate Data, and the Politics of Global Warming. The MIT Press. 552 p.

[69] National Center for Biotechnology Information (n.d.) GenBank Overview. Available: http://www.ncbi.nlm.nih.gov/genbank/. Accessed 2013 Apr 2.

[70] Regents of the University of Michigan (2011) ICPSR - Inter-university Consortium for Political and Social Research. Available: http://www.icpsr.umich.edu/icpsrweb/ICPSR/. Accessed 2013 Apr 2.

[71] Geeknet Inc. (2012) SourceForge: Download, Develop and Publish Free Open Source Software. Available: http://sourceforge.net/. Accessed 2013 Apr 2.

[72] Geeknet Inc. (2012) Freecode. Available: http://freecode.com/. Accessed 2013 Apr 2.

[73] Microsoft (2012) CodePlex: Open Source Project Hosting. Available: http://www.codeplex.com/. Accessed 2013 Apr 2.

[74] Wallis JC, Mayernik MS, Borgman CL, Pepe A (2010) Digital libraries for scientific data discovery and reuse: from vision to practical reality. Proceedings of the 10^th^ Annual Joint Conference on Digital Libraries. Gold Coast, Queensland, Australia: ACM Press. p. 333–340. doi:10.1145/1816123.1816173

[75] Mandell RA (2012) Researchers' Attitudes towards Data Discovery: Implications for a UCLA Data Registry. SSRN eLibrary. Available: http://papers.ssrn.com/sol3/papers.cfm?abstract_id=2129539. Accessed 2012 Aug 31.

[76] Mandell RA (2012) A New Tool for Managing and Discovering Research Data: Creating the UCLA Data Registry. Libraries in the Digital Age (LIDA) Proceedings. Zadar, Croatia, Vol. 12. Available: http://ozk.unizd.hr/proceedings/index.php/lida2012/article/view/59/43. Accessed 2013 Apr 2.

[77] Wynholds LA, Wallis JC, Borgman CL, Sands A, Traweek S (2012) Data, data use, and scientific inquiry: Two case studies of data practices. Proceedings of the 12th ACM/IEEE-CS joint conference on Digital Libraries. JCDL '12. New York, NY, USA: ACM. pp. 19–22. doi:10.1145/2232817.2232822

[78] Hamilton M (2004) CENS: New directions in wireless embedded networked sensing of natural andagricultural ecosystems. Converging Technologies for Agriculture and Evironment. Melbourne, Australia. Available: http://blueoakranchreserve.org/external_files/staff/Oliphant_Report.pdf. Accessed 2013 Apr 3.

[79] Borgman C, Wallis JC, Enyedy N (2006) Building Digital Libraries for Scientific Data: An Exploratory Study of Data Practices in Habitat Ecology. In: Gonzalo J, Thanos C, Verdejo MF, Carrasco RC, editors. Research and Advanced Technology for Digital Libraries. Berlin, Heidelberg: Springer Berlin Heidelberg, Vol. 4172. pp. 170–183. doi:10.1007/11863878_15

[80] Pepe A (2010) Structure and evolution of scientific collaboration networks in a modern research collaboratory United States – California: University of California, Los Angeles.

[81] Pepe A (2011) The relationship between acquaintanceship and coauthorship in scientific collaboration networks. Journal of the American Society for Information Science and Technology 62: 2121–2132 doi:10.1002/asi.21629

[82] Cragin MH, Palmer CL, Carlson JR, Witt M (2010) Data sharing, small science and institutional repositories. Philosophical Transactions of the Royal Society A: Mathematical, Physical and Engineering Sciences 368: 4023–4038 doi:10.1098/rsta.2010.0165 10.1098/rsta.2010.0165 20679120

[83] Sieber JE (1989) Sharing Scientific Data I: New Problems for IRBs. IRB: Ethics and Human Research 11: 4 doi:10.2307/3564184 11650286

[84] Sterling TD, Weinkam JJ (1990) Sharing scientific data. Communications of the ACM 33: 112–119 doi:10.1145/79173.79182

[85] Campbell EG (2002) Data Withholding in Academic Genetics: Evidence From a National Survey. JAMA: The Journal of the American Medical Association 287: 473–480 doi:10.1001/jama.287.4.473 11798369 10.1001/jama.287.4.473

[86] Gardner D, W.Toga A, Ascoli GA, Beatty ackson T, Brinkley JF, et al (2003) Towards Effective and Rewarding Data Sharing. Neuroinformatics 1: 289–296 doi:10.1385/NI:1:3:289 15046250 10.1385/NI:1:3:289

[87] Foster MW, Sharp RR (2007) Share and share alike: deciding how to distribute the scientific and social benefits of genomic data. Nature Reviews Genetics 8: 633–639 doi:10.1038/nrg2124 10.1038/nrg2124 17607307

[88] Bertzky M, Stoll-Kleemann S (2009) Multi-level discrepancies with sharing data on protected areas: What we have and what we need for the global village. Journal of Environmental Management 90: 8–24 doi:10.1016/j.jenvman.2007.11.001 18207633 10.1016/j.jenvman.2007.11.001

[89] Van House NA, Butler MH, Schiff LR (1998) Cooperative knowledge work and practices of trust ACM Press. pp. 335–343. Available: http://portal.acm.org/citation.cfm?doid=289444.289508. Accessed 2012 Aug 31.

[90] Pritchard SM, Carver L, Anand S (2004) Collaboration for Knowledge Management and Campus Informatics. University of California at Santa Barbara. 38 p. Available: http://www.library.ucsb.edu/informatics/informatics/documents/UCSB_Campus_Informatics_Project_Report.pdf. Accessed 2006 Jul 5.

